# The prevalence of subclinical hypothyroidism in a pre-diabetes population and an analysis of related factors

**DOI:** 10.1080/07853890.2023.2178668

**Published:** 2023-02-22

**Authors:** Xingyu Chang, Yaqi Wang, Yi Liu, Yanyu Shen, Jiaqing Feng, Qianqian Liu, Chenjun Jiang, Jing Yu, Xulei Tang, Gaojing Jing, Qianglong Niu, Songbo Fu

**Affiliations:** a The First Clinical Medical College, Lanzhou University, Lanzhou, China; b Department of Endocrinology, First Hospital of Lanzhou University, Lanzhou, China; c Gansu Province Clinical Research Center for Endocrine Disease, China

**Keywords:** Pre-diabetes, subclinical hypothyroidism, risk factors

## Abstract

**Background:**

To investigate the prevalence and related influencing factors of subclinical hypothyroidism (SCH) in a pre-diabetes (PreDM) population.

**Patients and methods:**

A multi-stage stratified cluster random sampling method was used to select the adult Han population in Gansu Province for investigation. General data and related biochemical indices were recorded and SPSS software was used for statistical analyses.

**Results:**

This study selected 2876 patients, including 548 with SCH and 433 with PreDM. In the PreDM population, the levels of thyroid stimulating hormone (TSH), serum phosphorus, TPOAb and TgAb in the SCH group were higher than those in the euthyroid group (*P* < 0.05). The level of TPOAb in females of SCH group was higher than that in males (*P* < 0.05). The positive rates of TPOAb and TgAb in females were higher than those in males in the total population and SCH population. The prevalence of SCH in the PreDM group under 60 was significantly higher than that in the normal glucose tolerance (NGT) group (26.02% vs. 20.40%, *χ*^2^ = 5.150, *P* < 0.05). We defined SCH as a TSH level of >4.20 mIU/L. Using this criterion, the prevalence of SCH in the total population of PreDM was higher than that in the NGT population (*χ*^2^ = 8.611, *P* < 0.05), the prevalence of SCH in the PreDM population generally showed an upward trend. However, we performed a separate analysis considering the accepted impact of age on TSH redefining SCH as TSH >8.86 mIU/L (for individuals over age 65). However, allowing for the expected rise in TSH levels in individuals over age 65, the prevalence of SCH in the elderly over 65 years of age decreased significantly (NGT population from 27.48% to 9.16%, PreDM population from 34.18% to 6.33%, *P* < 0.05). Logistic regression analysis showed that the risk factors for SCH in the PreDM population were female gender, fasting plasma glucose and TSH (all *P* < 0.05). Risk factors for SCH in the impaired fasting glucose (IFG) population were female gender, OGTT 2 h, TSH and TPOAb (all *P* < 0.05).

**Conclusion:**

The prevalence of SCH in the PreDM population not considering the known physiological increase in age related TSH was relatively high and was significant in female and the IFG population. However, the effect of age on these findings needs to attract more attention.

## KEY MESSAGES

The prevalence of subclinical hypothyroidism (SCH) in the pre-diabetic population was analysed by cross-sectional survey. There is a great deviation in the diagnosis of SCH in the elderly with physiologically increased thyroid stimulating hormone, which needs to be redefined.

## 1. Introduction

Pre-diabetes (PreDM) is a transitional state between normal glucose tolerance (NGT) and diabetes [1]. In recent years, with the rapid economic development and changes in dietary structure, the number of patients with PreDM has continued to increase. It is estimated that the number of patients worldwide will reach 470 million in 2030, and the prevalence rate of PreDM in China could reach as high as 35.2% [2,3]. Subclinical hypothyroidism (SCH) refers to the increase in serum thyroid stimulating hormone (TSH) levels while thyroid hormone levels remain normal. A study based on 31 provinces found that the prevalence of SCH in China reached 12.93% [4]. This condition is often ignored until it develops into clinical hypothyroidism and increases the risk of cardiovascular and other diseases [5]. Previous studies have found that abnormal glucose metabolism closely relates to the occurrence of SCH, but its mechanism has not yet been fully clarified, and relatively few studies have included PreDM populations [6]. Therefore, this study selected adult residents in Gansu Province to analyse the prevalence and characteristics of SCH in a PreDM population and explore the influence of related indicators and their relationship with TSH. Our findings provide a reference for the diagnosis of SCH in the Chinese population.

## 2. Materials and methods

### 2.1. Research subjects

#### 2.1.1. Selection method

A multi-stage stratified cluster random sampling method was used in Gansu Province. From 4 September 2016 to 1 February 2017, adult Han residents living in Lanzhou, Longnan, Dingxi, Baiyin and Linxia for more than 5 years were randomly selected. Following the procedure of ‘registration first and mobilization later’, the registered population at each site should be more than twice the size of the sample to meet the number of samples and the requirements of gender and age structure, and to prevent voluntary entry into the investigation queue by persons outside the site. A total of 2876 subjects were included, including 1463 males and 1413 females. The age range was 18–87 years, and the average age was 42.87 ± 14.99 years. Exclusion criteria: 1, severe heart, liver, renal insufficiency diseases, severe anaemia, or malignant tumours; 2, pregnant women or lactating women; 3, have taken drugs that interfere with blood lipids, blood pressure and thyroid function in the past 3 months. Such drugs included glucocorticoids, metoclopramide and propranolol.

### 2.2. Methods

#### 2.2.1. Clinical data

Under the guidance of professionals, the participants filled out the survey registration form and accurately recorded gender, age, height, weight, body mass index (BMI), waist circumference, heart rate, systolic blood pressure (SBP), diastolic blood pressure (DBP), family history of diabetes, history of hypertension, history of thyroid disease and other general conditions.

We obtained ethical approval and a letter of cooperation from the Medical Ethics Research Committee of the First Affiliated Hospital of China Medical University (AF-SOP-07-1.0-01), which was conducted in accordance with the Declaration of Helsinki [7].

#### 2.2.2. Biochemical indices

The blood lipid-related indexes: total cholesterol (TC, mmol/L), triglyceride (TG, mmol/L), high-density lipoprotein cholesterol (HDL-C, mmol/L) and low-density lipoprotein cholesterol (LDL-C, mmol/L); uric acid (UA, mmol/L), aspartate aminotransferase, alanine aminotransferase, blood calcium and serum phosphate (mmol/L) using test kit and biochemical analyser (BS-180, Mindray company). Fasting plasma glucose (FPG, mmol/L) and 2 h blood glucose after OGTT load (2 h PG, mmol/L) were determined by glucose oxidase method using test kit and biochemical analyzer (BS-180, Mindray Company). Glycosylated hemoglobin (HbA1c, %) using BioRad reagent, measured by VARIANT II (BioRad Company). Thyroid-stimulating hormone (TSH, mIU/L), Free thyroxine (FT4, pmol/L) anti-thyroid peroxidase antibody (TPOAb, 0–34IU/L) and anti-thyroid globulin antibody (TgAb, IU/L) using ICMA (Roche Company). Urinary iodine (UIC, g/L) was determined by inductively coupled plasma mass spectrometer (7700×, Agilent, USA).

#### 2.2.3. PreDM diagnostic criteria and grouping

According to the Guidelines for the Prevention and Treatment of Type 2 Diabetes in China (WHO1999) [1] the diagnostic criteria were as follows:

1. NGT: FPG < 6.1 mmol/L and 2 h PG < 7.8 mmol/L.
2. PreDM: (1) impaired fasting glucose (IFG): 6.1 mmol/≤FPG < 7.0 mmol/L and 2 h PG < 7.8 mmol/L. (2) impaired glucose tolerance (IGT): 7.8 mmol/L ≤ 2 h PG < 11.1 mmol/L and FPG < 6.1 mmol/L. (3) IGT combined with impaired fasting glucose (IFG + IGT): 6.1 mmol/L ≤ FPG < 7.0 mmol/L and 7.8 mmol/L ≤ 2h PG < 11.1 mmol/L. 3 Diabetes: FPG ≥ 7.0 mmol/L or 2 h PG ≥ 11.1 mmol/L.

#### 2.2.4. Subclinical hypothyroidism

In reference to the Guidelines for the Diagnosis and Treatment of Adult Hypothyroidism and An Age-Specific Serum Thyrotropin Reference Range for the Diagnosis of Thyroid Diseases in Older Adults: A Cross-Sectional Survey in China [8,9], the criteria are as follows: normal free thyroxine (FT4: 9.00–22.00 pmol/L) and free triiodothyronine (FT3: 3.1–6.8 pmol/L) levels. We defined SCH as a TSH level of >4.20 mIU/L. However, we performed a separate analysis considering the impact of age on TSH defining SCH as TSH >8.86 mIU/L (age > 65) statistical methods.

### 2.3. Statistical methods

SPSS software (version 25.0) was used for statistical analyses. Normal distribution measurement data are expressed as (*x* ± *s*). Two independent sample t-tests were used for comparisons between the two groups. Count data were described by frequency. The *χ*^2^ test was used to analyse the differences in prevalence between the two groups. A logistic regression analysis model was used to analyse the possible risk factors for PreDM and its different subtypes, with a test level of *α* = 0.05. Non-normal distribution data were expressed as median (median, M), 25th and 75th percentiles (P25 and P75, respectively). The Mann-Whitney U test was used for comparisons between the two groups. All of the comparison results were statistically significant (*P* < 0.05).

## 3. Results

### 3.1. Baseline data distribution of the survey population

Baseline data of the 2876 subjects, including region, educational level, occupation and annual household income, are shown in Table 1.

**Table 1.** Baseline data distribution of survey population.

Characteristics	Number of cases	Composition ratio
Area		
Urban	1597	55.53%
Rural	1279	44.47%
Education		
Illiteracy	346	12.03%
Primary school	282	9.81%
Junior high school	455	15.82%
Senior high school/technical secondary school	468	16.27%
Undergraduate/junior college	1252	43.53%
Postgraduate	73	2.54%
Profession		
Worker	790	27.47%
Farmer	1079	37.52%
Staff	664	23.09%
Housework	53	1.84%
Student	97	3.37%
Other	193	6.71%
Annual household income (1000 yuan)		
≤5	62	2.16%
5–10	178	6.19%
10–30	627	21.80%
30–50	570	19.82%
50–100	935	32.51%
>100	504	17.52%

### 3.2. Comparison of general data between SCH and euthyroid groups in the PreDM population

There are 433 subjects with PreDM in 2876 and the 426 subjects with PreDM were divided into euthyroid (excluding hyperthyroidism, subclinical hyperthyroidism, hypothyroidism and SCH) and SCH groups. Analysis of the general data of 426 patients with PreDM revealed that the levels of TSH, Serum phosphate, TPOAb and TgAb in the SCH group were higher than those in the euthyroid group (all *P* < 0.05). The levels of TSH, TPOAb and TgAb in females were higher than those in males in the euthyroid group, and the level of TPOAb in females was higher than that in males in PreDM group (all *P* < 0.05). The corresponding results are shown in Tables 2 and 3.

**Table 2.** Comparison of general data in SCH and euthyroid group among PreDM population.

Characteristics	Total (*n* = 426)			
	Euthyroid group *n* = 328	SCH group *n* = 98	*t*/*z*	*P*
Age	52.53 ± 14.33	49.95 ± 11.39	1.851	0.066
BMI (kg/m^2^)	24.80 ± 3.25	24.98 ± 3.06	–0.481	0.631
SBP (mmHg)	134.10 ± 18.87	136.31 ± 17.44	–1.031	0.303
DBP (mmHg)	81.23 ± 11.48	82.03 ± 11.95	–0.601	0.548
FPG (mmol/L)	5.65 ± 0.64	5.76 ± 0.65	–1.593	0.112
TG (mmol/L)	1.82 ± 1.13	2.01 ± 1.97	–1.205	0.229
TC (mmol/L)	4.65 ± 0.96	4.85 ± 0.94	–1.853	0.065
LDL-C (mmol/L)	2.89 ± 0.72	2.91 ± 0.67	–0.267	0.789
HDL-C (mmol/L)	1.46 ± 0.36	1.47 ± 0.34	–0.038	0.970
HbA1c(%)	5.53 ± 0.53	5.43 ± 0.47	1.655	0.099
TSH (mIU/L)	2.65 ± 1.31	6.87 ± 3.69	–11.094	<0.001
serum calcium (mmol/L)	2.19 ± 0.14	2.21 ± 0.10	–1.448	0.148
Serum phosphate (mmol/L)	0.99 ± 0.16	1.05 ± 0.19	3.176	0.002
UIC M (P25,P75)/(μg/L)	213.35 (148.95, 296.03)	193.35 (134.68, 301.33)	–0.386	0.700
UA M (P25,P75)/(μmol/L)	286 (227, 358.5)	268 (218, 336)	–1.743	0.081
TPOAb M (P25,P75)/(IU/L)	9.42 (6.75, 13.98)	10.58 (7.81, 16.18)	2.223	0.026
TgAb M (P25,P75)/(IU/L)	10.87 (10.00, 17.80)	14.69 (10.00, 22.79)	2.824	0.005

**Table 3.** Comparison of thyroid-related indicators in SCH and euthyroid group among PreDM population.

Characteristics	Total (*n* = 426)							
	Euthyroid group				SCH group			
	Male (*n* = 189)	Female (*n* = 139)	*t*/*z*	*P*	Male, *n* = 39	Female, *n* = 59	*t*/*z*	*P*
TSH (mIU/L)	2.52 ± 1.31	2.82 ± 1.29	–2.062	0.04	6.43 ± 2.40	7.15 ± 4.34	–0.944	0.348
TPOAb M (P25, P75)/(IU/L)	8.73 (6.52, 12.38)	11.47 (7.30,19.34)	3.938	<0.001	9.36 (7.61, 14.06)	12 (9.08,19.24)	2.341	0.019
TgAb M (P25, P75)/(IU/L)	10 (10.00,15.09)	12.64 (10.00,22.07)	3.498	<0.001	13.21 (10.00, 17.42)	15.48 (10.00,60.21)	1.488	0.137

### 3.3. Comparison of the prevalence of SCH in PreDM and its subtype population

This study included 2659 participants in the following cohorts: NGT (2226 individuals) and PreDM (433 individuals). This study also included 548 patients with SCH (231 males and 317 females). The prevalence of SCH in the PreDM population was 22.63% (98/433). Furthermore, the prevalence of SCH in females was higher than that in males in PreDM and IFG populations (28.92% vs. 17.03%, 46.51% vs. 15.91%, all *P* < 0.05). The prevalence of SCH in males compared with that in females were statistically significant (17.20% vs. 24.09%, P*<0.001, *χ*^2^ =19.273), and as shown in Table 4.

**Table 4.** Comparison of the prevalence of SCH in PreDM and its subtype population.

	Male		Female		P*	*χ* ^2^	Total	
	*n*	n-SCH (%)	*n*	n-SCH (%)			*n* (%)	n-SCH (%)
PreDM	229	39 (17.03)	204	59 (28.92)	0.002	9.639	433	98 (22.63)
IFG	44	7 (15.91)	43	20 (46.51)	0.003	9.124	87	27 (31.30)
IGT	150	26 (17.33)	137	31 (22.63)	0.188	1.733	287	57 (19.86)
IFG + IGT	35	6 (17.14)	24	8 (33.33)	0.151	2.062	59	14 (23.73)
NGT	1114	192 (17.24)	1112	258 (23.20)	<0.001	13.533	2226	450 (20.22)
*P* ^#^	0.890		0.076		–		0.276	
*χ* ^2^	0.019		3.158				1.188	
Total	1343	231(17.20)	1316	317(24.09)	<0.001	19.273	2659	548 (20.61)

*n*: Total number of subjects; n-SCH: number of patients with subclinical hypothyroidism; P*: Comparison between male and female patients with SCH; *P*^#^: Comparison between PreDM and NGT patients with SCH.

### 3.4. Distribution of SCH prevalence in different age groups based on differences in glucose metabolism

**Figure 1.**
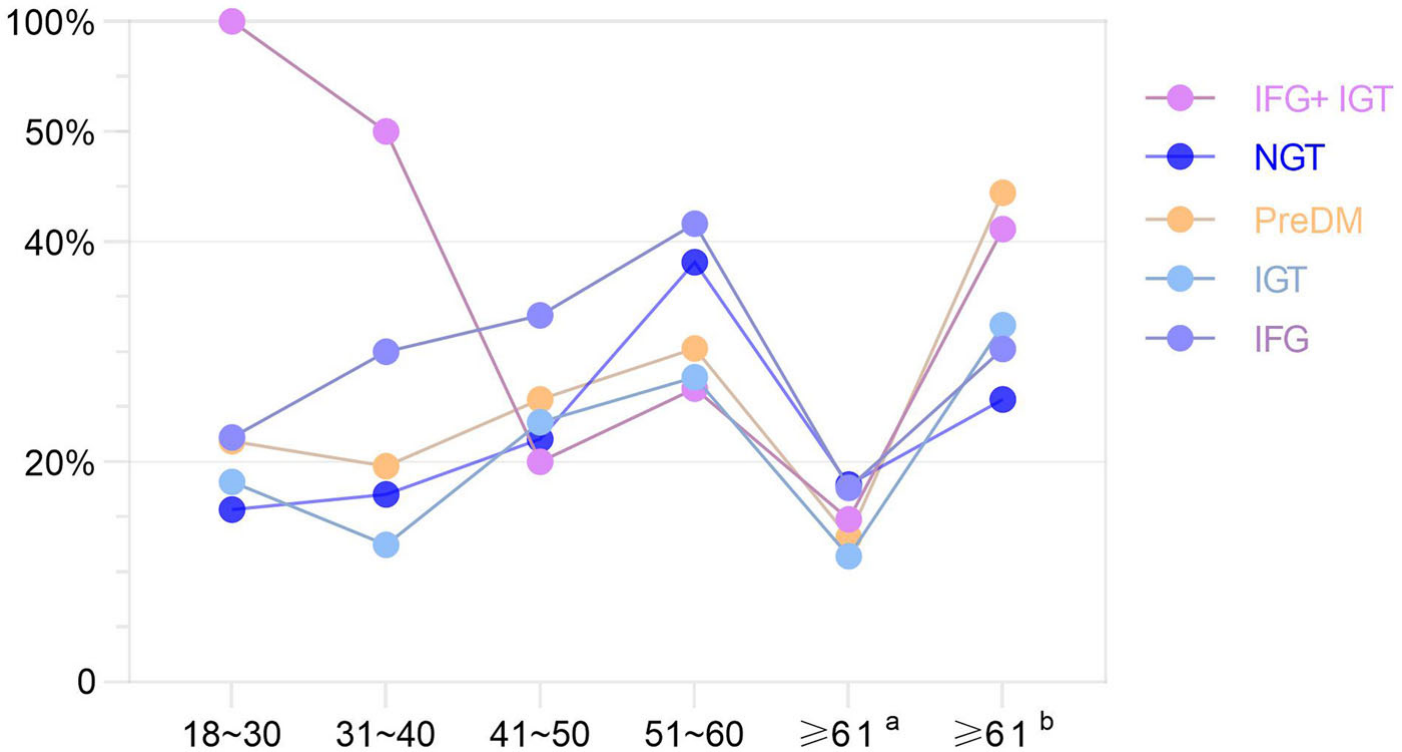
Distribution of SCH prevalence in different age groups under different glucose metabolism.

Total^a^ represents the consideration of the physiological increase of TSH in the elderly, the diagnostic criteria of SCH for people under 65 years old (TSH > 4.20 mIU/L, FT3 and FT4 are normal), and different diagnostic criteria for SCH for people over 65 years old (TSH > 8.86 mIU/L, FT3, FT4 normal) the prevalence of SCH. Total^b^ represents the prevalence of SCH based on the same diagnostic criteria for SCH (TSH > 4.20 mIU/L, normal FT3 and FT4) for all ages regardless of the effect of age on TSH. (1) The prevalence of SCH in the PreDM population under the age of 60 was higher than that in the NGT population (26.02% vs. 20.40%, *χ*^2^ =5.150, *P* < 0.05). (2) When considering the effect of age on TSH, there was no difference in the prevalence of SCH in the total PreDM population compared with the NGT population; when not considering the effect of age on TSH, the prevalence of SCH in the total PreDM population was higher than that in the NGT population (27.71% vs. 21.29%, *χ*^2^ = 8.611, *P* < 0.05). (3) Regardless of whether the effect of age on TSH was considered, the prevalence of SCH in the IFG population was higher than that in the NGT population (all *P* < 0.05). (4) Among the three PreDM subtypes, the prevalence of SCH in the IFG population was higher (31.03%, 39.51%). (5) Age-stratified analysis showed that the prevalence of SCH in the PreDM population showed an overall upward trend with an increase age (<60). (6) In the ≥61-year-old population, the prevalence of SCH in both NGT and PreDM populations was significantly higher (17.95%^a^ vs. 30.26%^b^, *χ*^2^=8.047; 13.16%^a^ vs. 32.45%^b^, *χ*^2^=12.058, all *P* < 0.05). In the population ≥65 years old, the prevalence of SCH in both NGT and PreDM populations was significantly higher compared with considering the effect of age on TSH without considering the effect of age on TSH (9.16%^a^ vs. 27.48%^b^, *χ*^2^=14.692; 6.33%^a^ vs. 34.18%^b^, *χ*^2^=18.966, all *P* < 0.05), as shown in Table 5 and Figure 1.

**Table 5.** SCH prevalence in different age groups under different glucose metabolism.

	NGT		PreDM		Prevalence of SCH in different subtypes of PreDM					
					IFG		IGT		IFG + IGT	
Age	*n*	n-SCH (*n,* %)	*n*	n-SCH (*n,* %)	*n*	n-SCH (*n,* %)	*n*	n-SCH (*n,* %)	*n*	n-SCH (*n,* %)
18–30	727	114 (15.68)	32	7 (21.88)	9	2 (22.22)	22	4 (18.18)	1	1 (100.00)
31–40	563	96 (17.05)	56	11 (19.64)	10	3 (30.00)	40	5 (12.50)	6	3 (50.00)
41–50	484	107 (22.11)	109	28 (25.69)	27	9 (33.33)	72	17 (23.61)	10	2 (20.00)
51–60	257	98 (38.13)	122	37 (30.33)	24	10 (41.67)	83	23 (27.71)	15	4 (26.67)
Total (18–60)	2031	415 (20.40)	319	83 (26.02)^#^	70	24 (34.29)^#^	217	49 (22.58)	32	10 (31.25)
≥61^a^	195	35 (17.95)	114	15 (13.16)	17	3 (17.65)	70	8 (11.43)	27	4 (14.81)
≥65^a^	131	12 (9.16)	79	5 (6.33)	13	1 (7.69)	49	0 (0.00)	17	4 (23.53)
Total^a^ (18–87)	2226	450 (20.22)	433	98 (22.63)	87	27 (31.03)^#^	287	57 (19.86)	59	14 (23.73)
*χ* ^2^		71.810		11.343		3.459		8.615		6.836
*P*		<0.001		0.023		0.484		0.071		0.145
≥61^b^	195	59 (30.26)	114	37 (32.45)	17	7 (41.18)	70	18 (25.71)	27	12 (44.44)
≥65^b^	131	36 (27.48)	79	27 (34.18)	13	5 (38.46)	49	14 (28.57)	17	8 (47.06)
Total^b^ (18–87)	2226	474 (21.29)	433	120 (27.71)^#^	87	32 (36.78)^#^	287	74 (25.78)	59	22 (37.29)^#^

*n*: Total number; n-TN: Number of patients with SCH; %: prevalence; *P*: Comparison of the prevalence of SCH among different ages; ^#^: Comparison of SCH between PreDM/IFG/IGT/IFG + IGT population and NGT population, *P* < 0.05.

### 3.5. Thyroid antibodies in different populations

**Figure 2.**
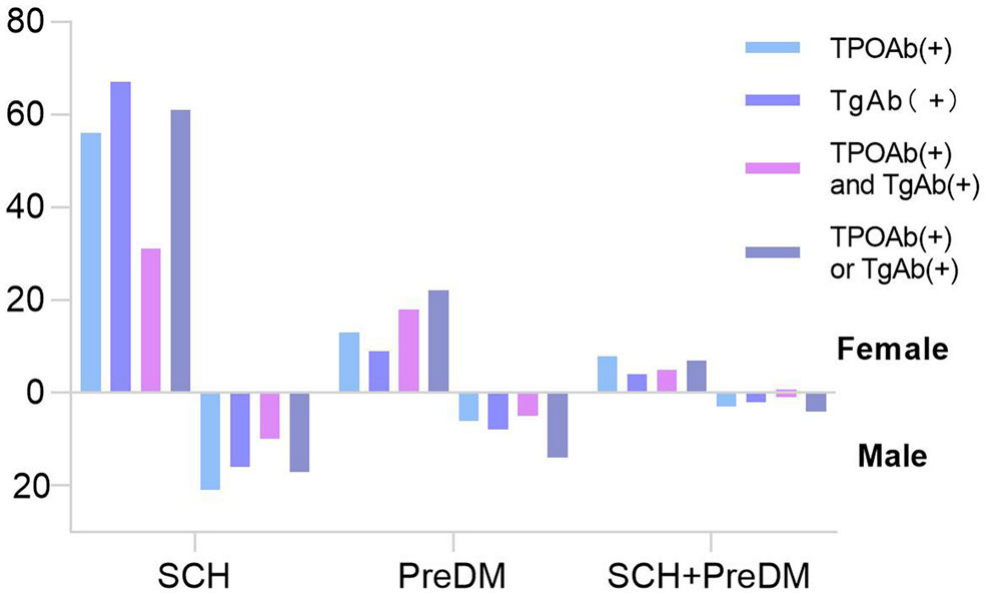
Thyroid antibodies in different populations.

In the total population and SCH population, the positive rate of thyroid-associated antibody in females was higher than that of males (all *P* < 0.05). In the PreDM population, the positive rate of both TPOAb (+)and TgAb (+) in females was higher than that of males (*P* < 0.05). There was no significant difference in the positive rate of thyroid-associated antibody between male and female in the SCH combined with PreDM, as shown in Table 6 and Figure 2.

**Table 6.** Thyroid antibodies in different populations.

Group	Total (2876)		*P*	*χ* ^2^	SCH (591)		*P*	*χ* ^2^	PreDM (433)		*P*	*χ* ^2^	SCH + PreDM(98)		*P*	*χ* ^2^
Gender	Male (1463)	Female (1413)			Male (249)	Female (342)			Male (229)	Female (204)			Male (39)	Female (59)		
TPOAb (+)	72	169	<0.001	46.387	21	56	0.005	8.018	6	13	0.057	3.621	3	8	0.368	0.811
TgAb (+)	61	194	<0.001	81.301	16	67	<0.001	13.330	8	9	0.623	0.241	2	4	0.739	0.111
TPOAb (+) and TgAb (+)	35	93	<0.001	29.665	10	31	0.017	5.688	5	18	0.002	9.458	1	5	0.232	1.427
TPOAb (+) or TgAb (+)	63	177	<0.001	63.503	17	61	<0.001	15.244	14	22	0.079	3.088	4	7	0.805	0.061
Both negative	1365	1143	<0.001	99.203	222	250	<0.001	23.103	210	164	0.001	11.728	34	47	0.336	0.926

### 3.6. Logistic regression analysis of risk factors for SCH in PreDM and IFG populations

In the PreDM and IFG populations, with or without SCH as the dependent variable, the independent variables were screened by single-factor analysis, and the independent variables with statistical significance were further evaluated in a multi-variate analysis. The results showed that the risk factors for SCH in the PreDM population were female gender, FPG, TSH (all *P* < 0.05). The risk factors for SCH in the IFG population were female gender, OGTT 2 h, TSH, TPOAb (all *P* < 0.05), as shown in Table 7.

**Table 7.** Logistic regression analysis of SCH risk factors in PreDM and IFG populations.

	PreDM			IFG		
	*P*	OR	95% Cl	*P*	OR	95% Cl
Gender	0.024	0.297	0.104–0.851	0.018	0.238	0.072–0.784
FPG	0.010	1.667	1.132–2.454	0.684	0.620	0.062–6.202
OGTT 2 h	0.217	0.891	0.742–1.070	0.043	1.894	1.020–3.514
TSH	<0.001	5.012	3.456–7.268	0.003	4.325	2.390–6.515
TPOAb	0.465	1.001	0.998–1.003	0.032	1.016	1.001–1.030

## 4. Discussion

Abnormal glucose metabolism and thyroid diseases are common factors that affect the health of Chinese residents. In the context of rapid social and economic development and population ageing, their prevention and treatment should be paid great attention [10]. SCH is closely related to PreDM, and the incidence rate of diabetes complicated with thyroid dysfunction is 12.5%–51.6%, with SCH being the most common [11].

The prevalence of SCH in the PreDM population under the age of 60 in Gansu was 26.02%, which was significantly higher than that in the NGT population (20.40%). However, the prevalence of SCH in the whole age group, considering that TSH levels increase physiologically with age (the SCH diagnostic criteria for people over 65-years-old consider TSH > 8.86 mIU/L alone), the prevalence of SCH in the total PreDM population is no higher than that in the NGT population. However, if the effect of age on TSH was not considered (the diagnostic criteria for SCH in all populations were TSH > 4.20 mIU/L), the prevalence of SCH in the total PreDM population was significantly higher than that in the NGT population (27.71% vs. 21.29%), it can be seen that there is a large floating difference in the prevalence of SCH in the elderly population. The diagnostic criteria of SCH for the elderly are still controversial. The review of Professor Biondi et al. showed that the serum TSH level of elderly patients may exceed the upper limit of the traditional reference range of 4–5 mU/L, which may lead to an overestimation of SCH in this age group. True prevalence of SCH in the above population [12]. This view is consistent with our study, which found that the prevalence of SCH was higher in people aged ≥65 years (NGT: 27.48%, PreDM: 34.18%) when the effect of age on TSH was not considered; considering the physiological increase in TSH caused by age, the prevalence of SCH in people aged ≥65 years was significantly lower (NGT: 9.16%, PreDM 6.33%). Some studies suggest that mildly elevated serum TSH in the elderly is not associated with increased morbidity and mortality [13], suggesting that we should update the diagnostic criteria of TSH according to the reference range of TSH in the elderly population in this region. When calculating the prevalence of SCH in the elderly, it is necessary to reconsider the threshold of TSH to avoid misdiagnosis of SCH in the population, so that the calculation of the prevalence of SCH is more reasonable, and it is helpful for the diagnosis and treatment of clinicians. However, in either case, the prevalence of SCH in Gansu PreDM population was much higher than that in Egypt (16.30%) [14] and Beijing (9.72%) [15], which may be related to many factors such as geographical environment and living habits (iodine consumption) in Gansu. At the same time, we found that among the three subtypes of PreDM, the prevalence of IFG population was slightly higher, and regardless of whether the effect of age on TSH was considered, the prevalence was higher than that of SCH in the NGT population.

In PreDM population, we found that the levels of TSH, TPOAb and TgAb in SCH group were higher than those in the euthyroid group, which may be because people with abnormal glucose metabolism are more prone to autoimmune thyroiditis (AIT) and thyroid cell destruction. There is increasing evidence that the imbalance of thyroid hormones and antibodies is related to the pathogenesis of type 1 diabetes [16]. Whether it is related to type 2 diabetes needs further study. At the same time, the leptin levels in patients with abnormal glucose metabolism is higher, which may affect the hypothalamus–pituitary–thyroid axis *in vitro* and *in vivo* through the Janus activated kinase (JAK)-2/signal transduction and transcription activation (STAT) 3 pathway, thereby stimulating the synthesis of TSH and affecting thyroid function [10]. Further gender-stratified analyses of the PreDM population found that the prevalence of SCH in females was always higher than that in males, in agreement with the findings of El-Eshmawy et al. [14] who also observed that SCH in PreDM was more common in females. The logistic regression analysis model showed that females with PreDM have a higher risk of developing SCH, which suggests that females are at greater risk for SCH and need more clinical attention.

AIT is the most common human organ-specific autoimmune disease, Hashimoto’s thyroiditis accounts for the vast majority. Its incidence is ∼10% in the general population, with a male to female ratio of 1:10. TgAb and TPOAb are the serum markers of AIT [17]. Our research found that, in the gender comparison of thyroid antibody positive rate, the positive rate of thyroid-related antibody in females was higher than that in males in the general population and in the SCH population, that is, the prevalence of AIT in females was higher. Research found that patients with abnormal glucose metabolism had higher thyroid autoantibody (TPOAb and TgAb) positive rate compared with the control group [18]. Most studies believe that the prevalence of AIT in females is high, the high positive rate of thyroid-related antibodies lead to an increase in the prevalence of SCH compared with males. However, we found that there was no difference in the positive rate of thyroid antibodies between females and males in the population of PreDM complicated with SCH. We speculate that the reason why the prevalence of SCH in the PreDM population is higher than that in the NGT population may be related to dysglycemia and diabetes-related AIT disease [19]. Abnormal glucose metabolism may interfere with thyroid metabolism by disturbing the level of thyroid hormone in plasma [20]. In addition, it may also be due to the small sample size of this population and certain limitations in this study. Our analysis is cross sectional and not longitudinal. And self-limited thyroiditis could account for the finding of elevated TSH in a subfraction of the cases in our report.

It is necessary to comprehensively pay attention to the relationship between glucose metabolism and thyroid hormone levels. However, our research still has some shortcomings, for example, the accuracy of the measurement of TSH level at a single time point to reflect the real situation of thyroid function level is a question worth considering. However, in view of the actual situation and ethical requirements, we have not been able to measure it multiple times, which is what we need to pay attention to next. Second, with the increase of age, the prevalence of glucose metabolism and thyroid diseases will increase. However, due to the interaction between the two diseases, we cannot determine the causal relationship. If we consider that TSH level increases physiologically with age, then the conclusion that TSH of people with abnormal glucose metabolism is higher than that of people with NGT needs further research.

## 5. Conclusion

In conclusion, the prevalence of SCH is high in the PreDM population in Gansu Province, and further subdivision of SCH severity may provide the next research direction for studying the relationship between SCH and PreDM. Improvement of blood glucose levels in PreDM patients, early SCH screening and corresponding interventions may have a positive effect on reducing the prevalence of SCH in PreDM patients.

## Data Availability

The data that support the findings of this study are available from the corresponding author.
